# Xenin-25 improves indomethacin-induced acute gastric injury in rats

**DOI:** 10.1007/s00424-026-03186-9

**Published:** 2026-06-09

**Authors:** Sevil Arabacı Tamer, Gamze Güney Eskiler, Feriha Ercan, Berrak Ç. Yeğen

**Affiliations:** 1 Department of Physiology, Sakarya University, Sakarya, Türkiye; 2 Department of Medical Biology, Sakarya University, Sakarya, Türkiye; 3 Department of Histology and Embryology, Marmara University, İstanbul, Türkiye; 4 Department of Physiology, Marmara University, İstanbul, Türkiye

**Keywords:** Gastric ulcer, Xenin-25, Inflammation, Apoptosis, Vagal afferent fibers

## Abstract

Putative gastroprotective effects of xenin-25 were investigated in an indomethacin-induced acute ulcer model. Male Sprague-Dawley rats were randomly assigned to control, saline-treated ulcer, xenin-25-treated ulcer groups. Gastric ulcer was induced with indomethacin (25 mg/kg, subcutaneously) and xenin-25 (0.2, 2, 20 µg/kg) or saline was given subcutaneously immediately after and at 2 h following indomethacin injection. In order to investigate whether the effects of xenin-25 depend on vagal afferent fibres, two additional groups underwent vagal afferent denervation (VAD) by bilateral perivagal capsaicin application (1%). Following a 2-week recovery, rats with VAD were induced with ulcer, after which they received either xenin-25 (2 µg/kg) or saline. All animals were sacrificed 4 h after ulcer induction, and gastric tissues were collected for biochemical, molecular, and histopathological analyses. Indomethacin administration resulted in significant increases in malondialdehyde and myeloperoxidase levels, accompanied by glutathione depletion, upregulation of *nuclear factor-κB* and *Bax*, and downregulation of *Bcl2* expression. Xenin-25 treatment markedly attenuated oxidative stress, inflammatory responses, apoptotic markers, and histopathological gastric damage. Xenin-25-induced reductions in lesion length, malondialdehyde, and myeloperoxidase levels were abolished in VAD rats, but all the other anti-inflammatory and anti-apoptotic effects of Xenin-25 were preserved even in the absence of vagal afferents. Xenin-25 exhibits gastroprotective effects in acute gastric injury through its antioxidant, anti-inflammatory, and anti-apoptotic actions, which are independent of vagal afferent fibers.

## Introduction

Peptic ulcer disease (PUD), predominantly affecting the stomach and proximal duodenum, has a reported lifetime prevalence of approximately 5–10% and an annual incidence of 0.1–0.3% in Western countries [1]. It arises from an imbalance between aggressive factors (e.g. gastric acid, reactive oxygen species (ROS), and inflammatory mediators), and the protective mechanisms of the mucosa [2, 3]. Non-steroidal anti-inflammatory drugs (NSAIDs), such as indomethacin, are a common etiological agent of gastric injury [4, 5]. They induce this damage not only by inhibiting cyclooxygenase (COX) and reducing prostaglandin synthesis but also by promoting oxidative stress, triggering inflammatory responses, and activating apoptotic pathways in gastric epithelial cells [6].

The vagus nerve innervates various internal organs and controls gastrointestinal motility and secretions [7]. Recent studies have established that the gastroprotective effects mediated by the vagus nerve involve the activation of cholinergic pathways, which promote the release of gastric prostaglandins and nitric oxide [8, 9]. Capsaicin-sensitive vagal afferent fibers protect the mucosa by modulating local blood flow, immune responses, and epithelial homeostasis [10, 11]. Therefore, understanding whether candidate therapeutic agents exert their protective effects via vagal afferent pathways is crucial for elucidating their mechanisms of action.

Gastrointestinal peptides are critical regulators of mucosal defense mechanisms and offer promising therapeutic options for preventing and treating gastric injuries. Xenin-25, which is a 25-amino-acid peptide hormone secreted in response to food intake mainly by duodenal K-cells along with glucose-dependent insulinotropic polypeptide (GIP; gastric inhibitory polypeptide) [12]. Xenin-25 exerts a range of physiological functions partly via the activation of neurotensin receptor 1 (NTSR1) [13, 14], and modulates energy homeostasis [15] and lipid metabolism [16], suppresses food intake [17] and gastrointestinal motility [13, 18], stimulates both exocrine and endocrine pancreatic functions [19], and regulates anion secretion in the ileum [20], In high-fat diet-fed mice it was reported that administering a Xenin-25 analog suppressed Nuclear Factor kappa B(NF-κB) and toll like receptor 4 (TLR4) signaling in the brain and reduced oxidative stress, suggesting its anti-inflammatory potential [21]. However, studies exploring the relationship between Xenin-25 and inflammation remain limited. Although Xenin-25 is hypothesized to exert protective effects against ulcer-induced inflammatory and oxidative injury, its impact on gastric ulceration has not yet been investigated in the existing literature.

This study aimed to evaluate the gastroprotective effects of Xenin-25 in a rat model of indomethacin-induced acute gastric ulceration. A secondary objective was to assess whether these putative gastroprotective effects of Xenin-25 are mediated via capsaicin-sensitive vagal afferent fibers.

## Materials and methods

### Animals and experimental procedure

Male Sprague–Dawley adult rats (12 weeks old, 230–270 g) were obtained from the Sakarya University Experimental Animal Center. Rats were housed under controlled conditions (22 ± 2 °C, 12 h light/dark cycle) with free access to food and water. All experimental procedures were conducted in accordance with national guidelines and approved by the Sakarya University Animal Experiments Ethics Committee (approval number: 01, date: 04.01.2023).

The rats were randomly divided into seven groups (*n* = 7 per group) as control, ulcer, ulcer treated with Xenin-25 (0.2, 2, or 20 µg/kg), ulcer with vagal afferent denervation (VAD), and ulcer with VAD plus Xenin-25 (2 µg/kg). In order to induce an acute gastric ulcer, indomethacin (25 mg/kg in 5% NaHCO3; Sigma-Aldrich, Missouri, USA) was injected subcutaneously (s.c.) following a 24-hour fasting period (with free access to water), while the control group received vehicle (5% sodium bicarbonate (NaHCO₃), 1 mL/kg, s.c.). Indomethacin-induced ulcer is widely used in rats to investigate acute gastric ulcerogenesis and to evaluate potential therapeutic agents [22, 23]. Xenin-25 (Phoenix Pharmaceuticals, Inc.) was dissolved in saline before administration. A dose–response analysis was conducted to evaluate the therapeutic efficacy of Xenin-25 in the treatment of gastric injury. The selected doses of Xenin-25 were based on a previous report [24]. Immediately after ulcer induction, rats were administered s.c. with either Xenin-25 at 0.2, 2, or 20 µg/kg doses or saline (1 ml/kg). Injections were repeated 2 h after the initial treatment. In the VAD group, fourteen days after the denervation procedure, indomethacin was injected to induce ulcer, and the effective dose (2 µg/kg) of Xenin-25 was administered subcutaneously based on the results of the dose-response analysis.

Four hours after administration of indomethacin or vehicle, all rats were anesthetized (xylazine, 10 mg/kg, and ketamine, 50 mg/kg, intraperitoneally (i.p.), and blood was collected via cardiac puncture. Stomachs were opened along the lesser curvature for macroscopic evaluation. Serum samples were separated for biochemical analysis, and gastric tissues were collected for biochemical, molecular and histological examinations.

### Peri-vagal capsaicin-induced vagal afferent denervation

Vagal afferent denervation was performed via perivagal application of 1% capsaicin (Sigma-Aldrich), as previously described [25]. Following anesthesia (xylazine 10 mg/kg and ketamine 50 mg/kg, i.p.), right and left cervical vagus nerves were exposed and gently isolated from surrounding tissues under an operating microscope. A freshly prepared capsaicin solution (10% Tween 80, 10% ethanol, and 80% physiological saline) was applied directly to each nerve using a small cotton piece for 30 min, then rinsed with saline, and the incision was closed. Rats were monitored for 14 days postoperatively, and the effectiveness of vagal afferent denervation was assessed using the eye-wipe test. A drop of ammonium hydroxide (NH₄OH, 1% ) solution was instilled into the eye, and the presence of wiping movements was observed. Having no eye-wiping response verified the impairment of corneal afferents [38]. The same surgical procedure was performed in the control group as well as in the ulcer groups without VAD, but instead of capsaicin vehicle (10% Tween 80, 10% ethanol, and 80% saline) was applied onto the vagus nerve.

### Macroscopic evaluation of gastric tissue

For macroscopic assessment, the length of each gastric lesion was measured and expressed in millimeters [26]. Additionally, gastric injury was scored according to the following criteria (maximum score = 6): **0** = no visible damage;**1** = presence of blood in the gastric lumen;**2** = pinpoint erosions;**3** = 1–5 small erosions (< 2 mm);**4** = more than five small erosions (< 2 mm);**5** = 1–3 extensive erosions (> 2 mm);**6** = more than three extensive erosions (> 2 mm).

### Assessment of oxidative stress markers in gastric tissue

Gastric tissue samples were homogenized on ice in trichloroacetic acid (TCA) solution using a homogenizer (MTOPS SR30, Korea). The homogenates were centrifuged at 3000 rpm for 10 min at 4 °C, and the supernatants were collected for biochemical analysis. Lipid peroxidation in gastric tissue samples was evaluated by measuring malondialdehyde (MDA) levels based on thiobarbituric acid reactive substances (TBARS) formation. The results were then expressed as nanomoles of MDA per gram of tissue. Glutathione (GSH) levels were determined using a modified Ellman’s method. The results were presented in micromoles per gram of tissue [27].

### Measurement of myeloperoxidase (MPO) activity in gastric tissue

Myeloperoxidase (MPO) is an enzyme predominantly found in the azurophilic granules of polymorphonuclear leukocytes and is widely used as a biochemical marker for neutrophil infiltration in tissues [28]. Gastric MPO activity was assessed spectrophotometrically based on the hydrogen peroxide (H₂O₂)-dependent oxidation of o-dianisidine dihydrochloride. The change in absorbance was measured at 460 nm. MPO activity was expressed as units per gram of tissue [27].

### Determination of TNF-α, IL-6, and caspase-3 levels via enzyme-linked immunosorbent assay

Gastric mucosa was homogenized in ice-cold PBS with protease inhibitors. Supernatants and serum samples were used to measure TNF-α, IL-6, and caspase-3 levels using commercially available rat ELISA kits (E0764Ra, E0135Ra, and E1648Ra, respectively, BT-Lab, China), following the manufacturer’s protocols. According to the manufacturer’s instructions, all biochemical measurements in gastric tissue were normalized to total protein content, which was determined using a protein assay kit (E-BC-K318-M, Elabscience).

### Real-time PCR evaluation of gastric NF-κB and apoptosis-related genes

Gastric tissue homogenization was performed using a tissue homogenizer (MTOPS SR30, Korea). According to the manufacturer’s protocol, total RNA was extracted from the samples using the PureLink™ RNA Mini Kit (Invitrogen, USA). The concentration and purity of RNA were assessed with the Qubit™ 4 Fluorometer (Thermo Fisher Scientific, Waltham, MA, USA). Complementary DNA (cDNA) was synthesized from 1 µg of total RNA using the High-Capacity cDNA Reverse Transcription Kit (Thermo Fisher Scientific, Waltham, MA, USA). Quantitative real-time PCR (RT-qPCR) was conducted using the StepOnePlus™ Real-Time PCR System (Applied Biosystems, Foster City, CA) with TaqMan™ Gene Expression Assays specific for *NF-κB*,* Bax*, and *Bcl2*, with *β-actin* serving as the endogenous control. All reactions were performed in triplicate. Gene expression levels were expressed as fold changes relative to the control group.

### Histopathological evaluation

The gastric tissue specimens were fixed in 10% neutral-buffered formalin and processed according to standard paraffin embedding procedures. Section  (5 μm) were prepared and stained with hematoxylin and eosin for general histological examination. A blinded histopathologist (FE) evaluated at least five representative fields per section using a light microscope (Olympus BX51, Tokyo, Japan). Histological alterations were scored semi-quantitatively on a scale from 0 to 3 for each parameter, with a maximum total score of 12. The scoring criteria included: surface epithelial loss, focal mucosal necrosis, congestion and hemorrhage, glandular degeneration, and inflammatory cell infiltration. Severity was graded as follows: 0 = none, 1 = mild, 2 = moderate, 3 = severe.

### Statistical analysis

All data are presented as mean ± SEM. For parametric data, statistical analysis was performed using one-way or two-way ANOVA, followed by Tukey’s post hoc test (GraphPad Prism 8.3.0). Nonparametric data, including histopathological and macroscopic lesion scores, were analyzed using the Kruskal–Wallis test followed by Dunn’s multiple-comparison test. Student’s t test was used to compare two groups. A p-value of < 0.05 was considered statistically significant.

## Results

### Macroscopic evaluation of gastric lesions

**Fig. 1:**
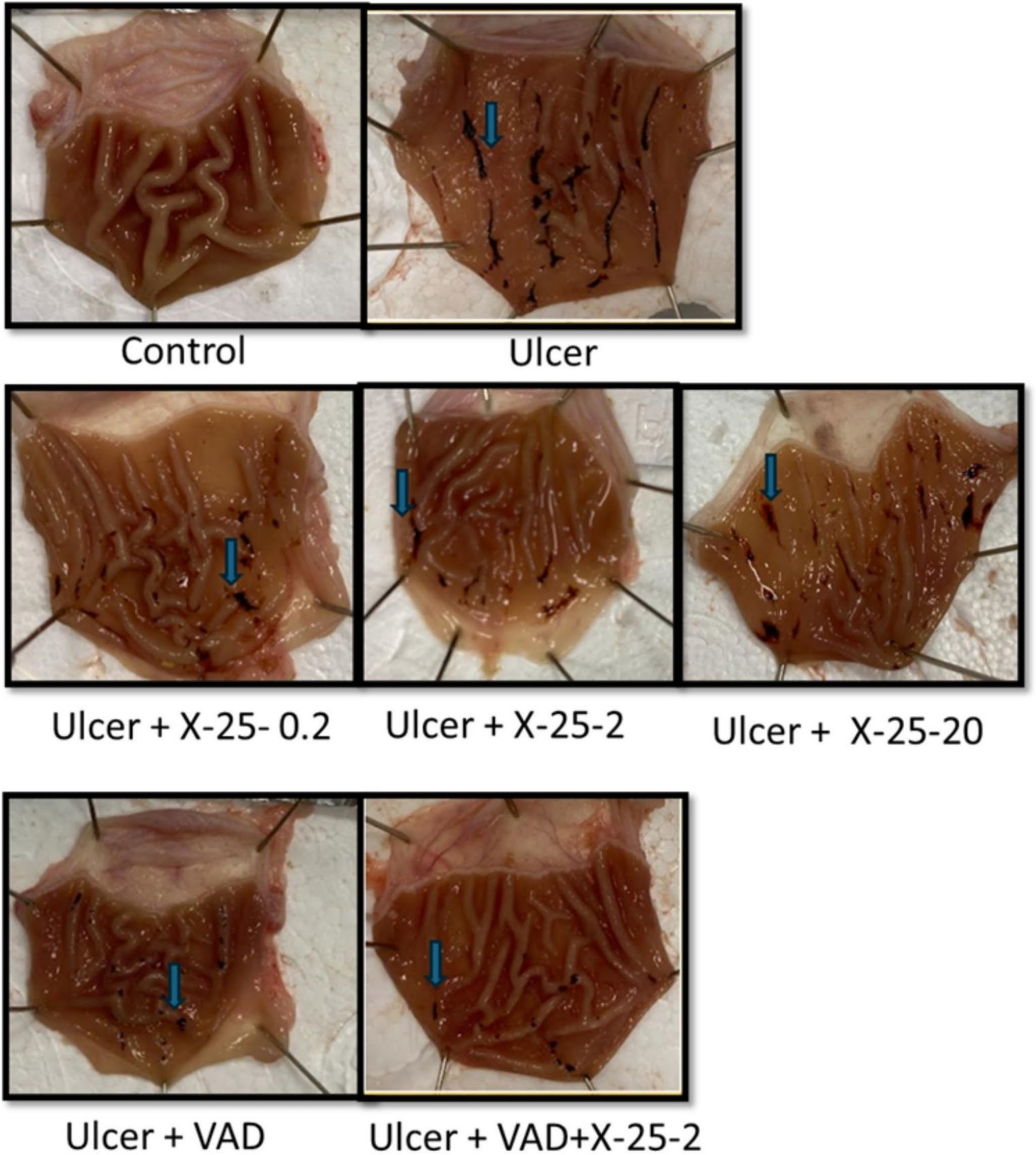
Representative photographs of the gastric mucosa in the experimental groups. VAD: Vagal afferent denervation with capsaicin, X-25: Xenin-25. The blue arrow represents the ulcer area

**Fig. 2:**
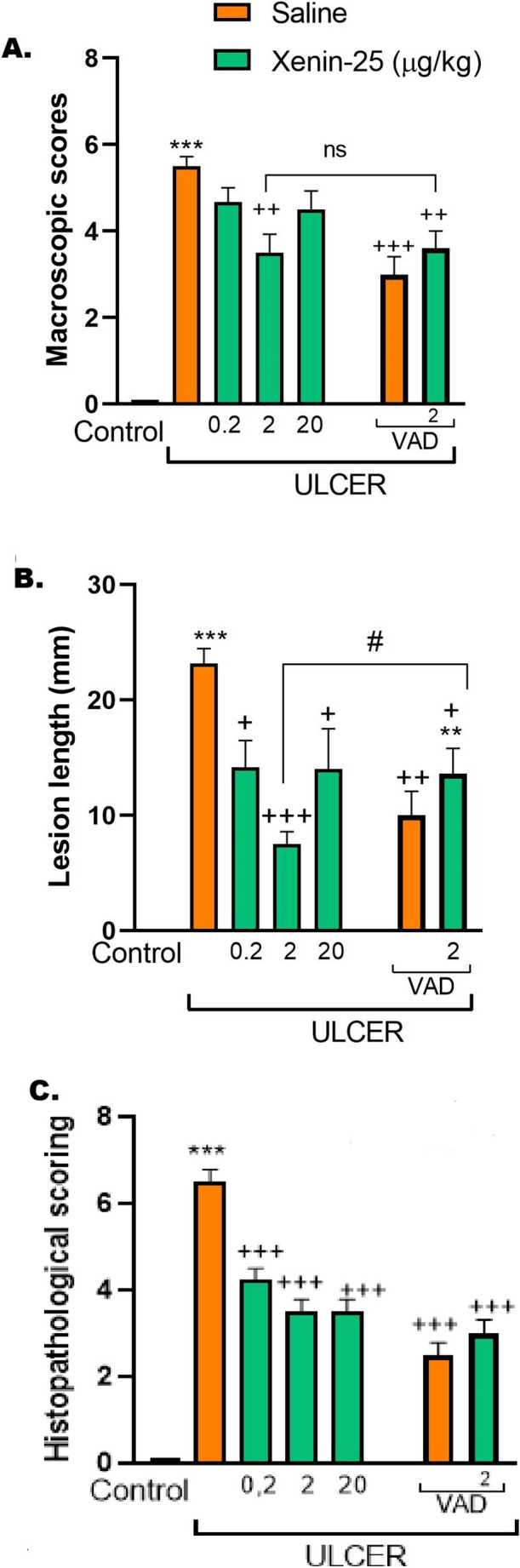
Macroscopic score (**A**), lesion lengths (**B**), and histopathological scores (**C**) of experimental groups. *n* = 7 per each group. ***p < 0.001 vs. control; +*p* < 0.05, ++*p* < 0.01, +++*p* < 0.001 vs. ulcer group. # *p* < 0.05 vs. Ulcer treated with a dosage of xenin-25 at 2 µg/kg. VAD: Vagal afferent denervation with capsaicin, X-25: Xenin-25

As shown in Fig. 1, control stomachs exhibited an intact mucosal surface with no visible lesions. In contrast, indomethacin-induced ulcer groups displayed pronounced longitudinal hemorrhagic streaks (blue arrows) across the gastric mucosa. On the other hand, xenin-25 treatment significantly improved the appearance of the gastric mucosa. Macroscopic and microscopic scores, as well as lesion lengths, were elevated compared to controls (*p* < 0.001; Fig. 2). Compared with the saline-treated ulcer group, macroscopic and histopathological scores, as well as lesion lengths (*p* < 0.05 − 0.001), were significantly lower in the xenin-25-treated ulcer groups, with the effect highly evident, especially at the middle dose (2 µg/kg).

### Histopathological findings

**Fig. 3:**
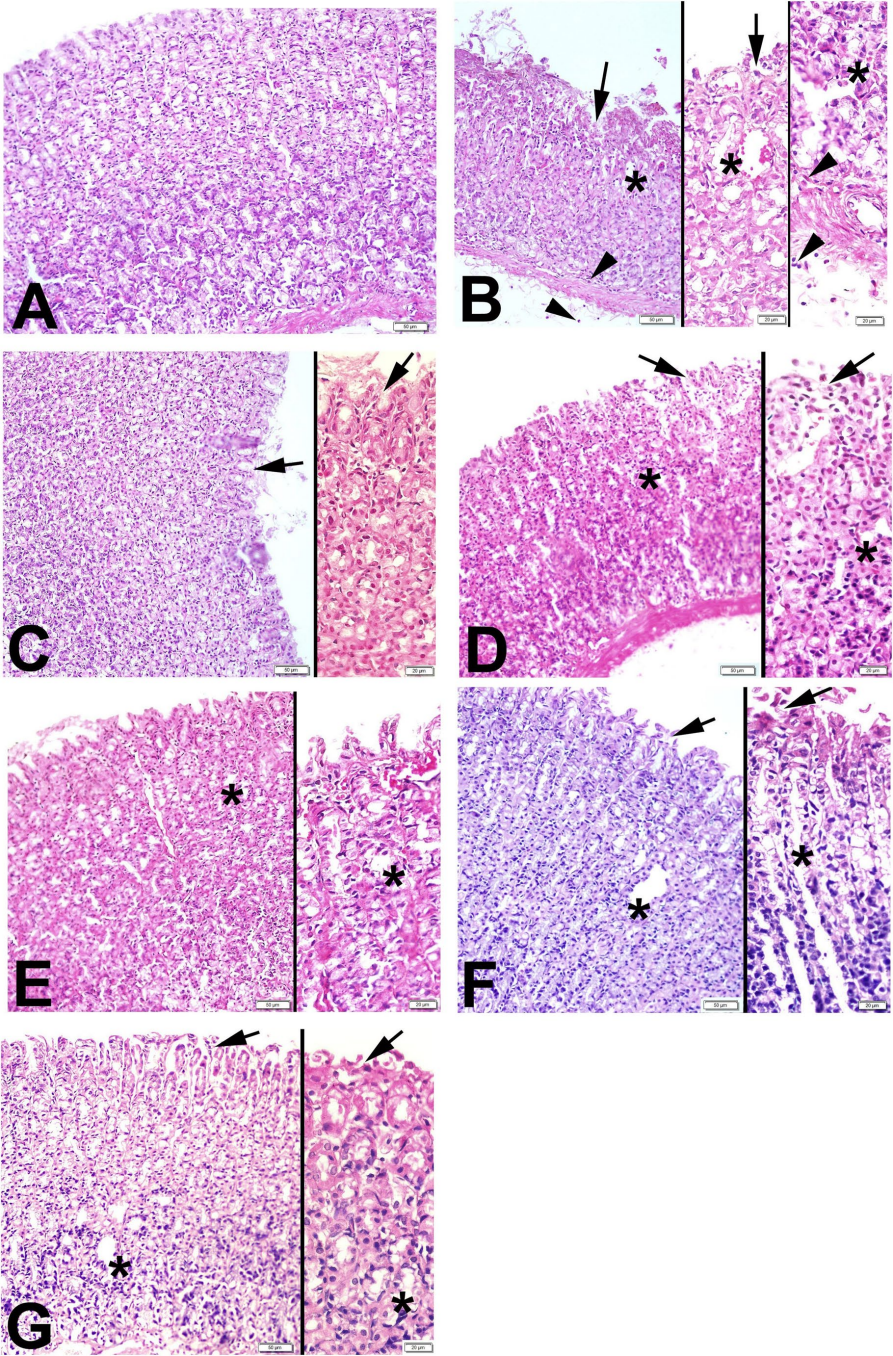
Representative light micrographs of experimental groups. Regular gastric mucosa is seen in the control group (**A**). Severe degeneration of surface (arrow) and glandular epithelium (*) and inflammatory cell infiltration (arrowhead) are seen in the indomethacin applied group (**B**). Degeneration of the surface (arrow) and glandular epithelium (*) was observed in the following groups: indomethacin plus Xenin-25 at 0.2 mg/kg (**C**), 2 mg/kg (**D**), 20 mg/kg (**E**), and indomethacin plus both VAD and Xenin-25 at 20 mg/kg (**F**). Hematoxylin and eosin staining. Original magnification: 200x, insets in B-G: 400x, scale bar: 50 μm, insets in **B**-**G**: 20 μm

In parallel with the macroscopic evidence, histopathological assessments revealed normal mucosal and submucosal morphology in the control group (Fig. 3). Severe degeneration of the mucosa, characterized by desquamated surface epithelium, degenerated glandular epithelium, mucosal bleeding, and inflammatory cell infiltration, was observed in the indomethacin-induced ulcer group. These changes were consistent with the markedly elevated histopathological scores (*p* < 0.001, Fig. 2). Mild to moderate mucosal degeneration with desquamated surface mucous cells, dilated glandular structures, inflammatory cell infiltration, and lower scores were observed in all doses of Xenin-25 (0.2, -2, and − 20 µg/kg) compared to the ulcer group (*p* < 0.001).

### Changes in the gastric oxidative stress markers

**Fig. 4:**
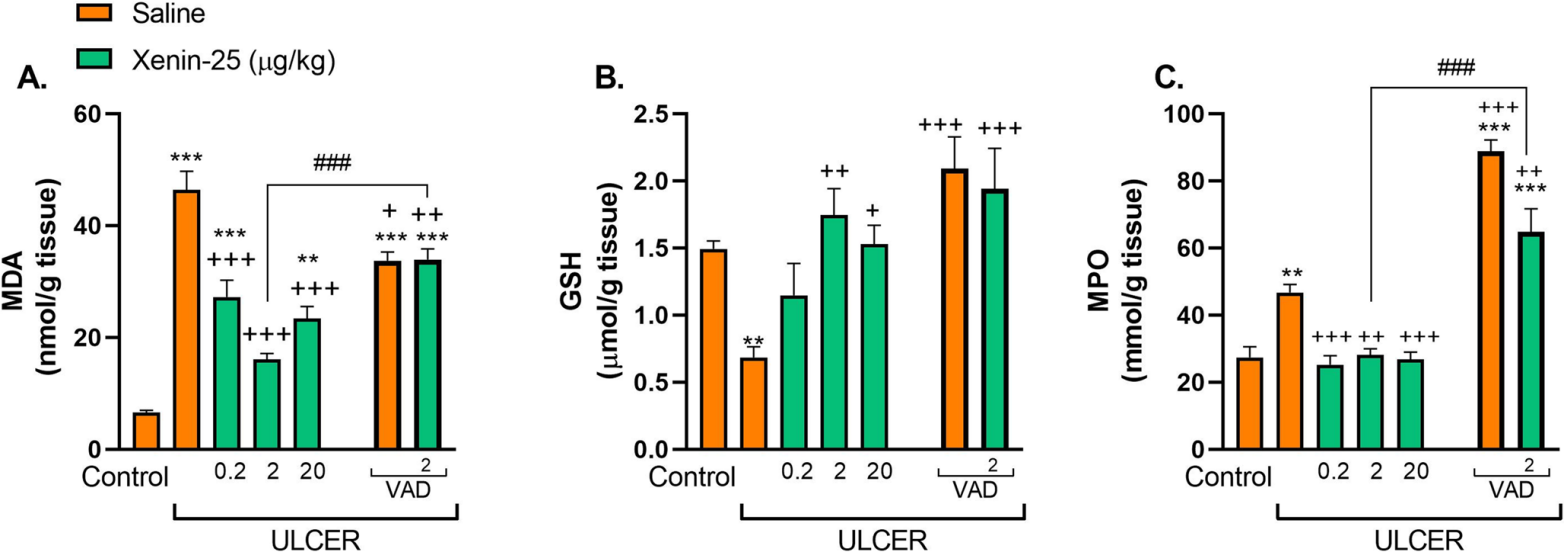
Effects of Xenin-25 on gastric oxidative stress markers in the indomethacin-induced ulcer rat model. *n* = 7 per each group. Gastric (**A**) malondialdehyde (MDA), (**B**) glutathione (GSH), and (**C**) myeloperoxidase (MPO) activity levels. **p < 0.01, ***p < 0.001, vs. control group; +*p* < 0.05, ++*p* < 0.01, +++*p* < 0.001 vs. ulcer group. ### *p* < 0.001 vs. X-25 at the 2 µg/kg dose-applied ulcer group. VAD: Vagal afferent denervation with capsaicin, X-25: Xenin-25

Compared to the control group, MDA and MPO levels were markedly elevated (*p* < 0.01–0.001), whereas GSH content was significantly reduced (*p* < 0.01) in the gastric tissue of the ulcer group (Fig. 4). Xenin-25 treatment reversed these changes, with the most pronounced effects observed at the 2 µg/kg dose (*p* < 0.05 − 0.001).

### Alterations in gastric and serum inflammatory markers

**Fig. 5:**
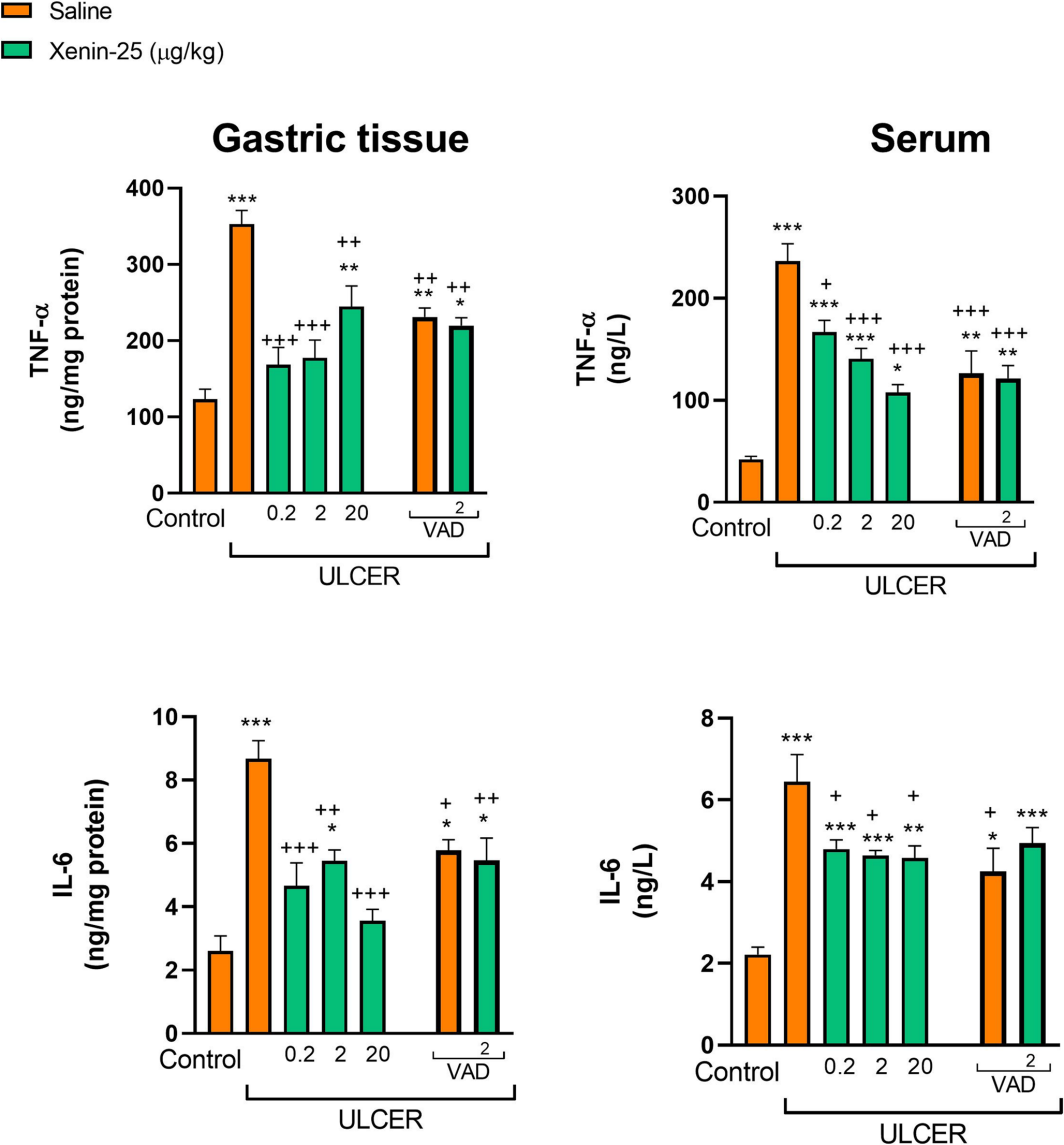
Effects of Xenin-25 on gastric and serum levels of TNF-α and IL-6 in the indomethacin-induced ulcer rat model. *n* = 7 per each group. *p < 0.05, **p < 0.01, ***p < 0.001 vs. control; +*p* < 0.05, ++*p* < 0.01, +++*p* < 0.001 vs. ulcer group. VAD: Vagal afferent denervation with capsaicin, X-25: Xenin-25

Ulcer induction, compared to the control group, resulted in significant increases in TNF-α and IL-6 levels in both serum and gastric tissue samples (*p* < 0.001, Fig. 5). Xenin-25 administration reduced these elevations compared with the saline-treated ulcer group (*p* < 0.05 − 0.001). Xenin-25 significantly reduced cytokine levels in ulcerated rats with intact vagal innervation compared to the saline-treated ulcer group (*p* < 0.05 − 0.001).

### Regulation of NF-κB activation and expression of apoptotic genes

**Fig. 6:**
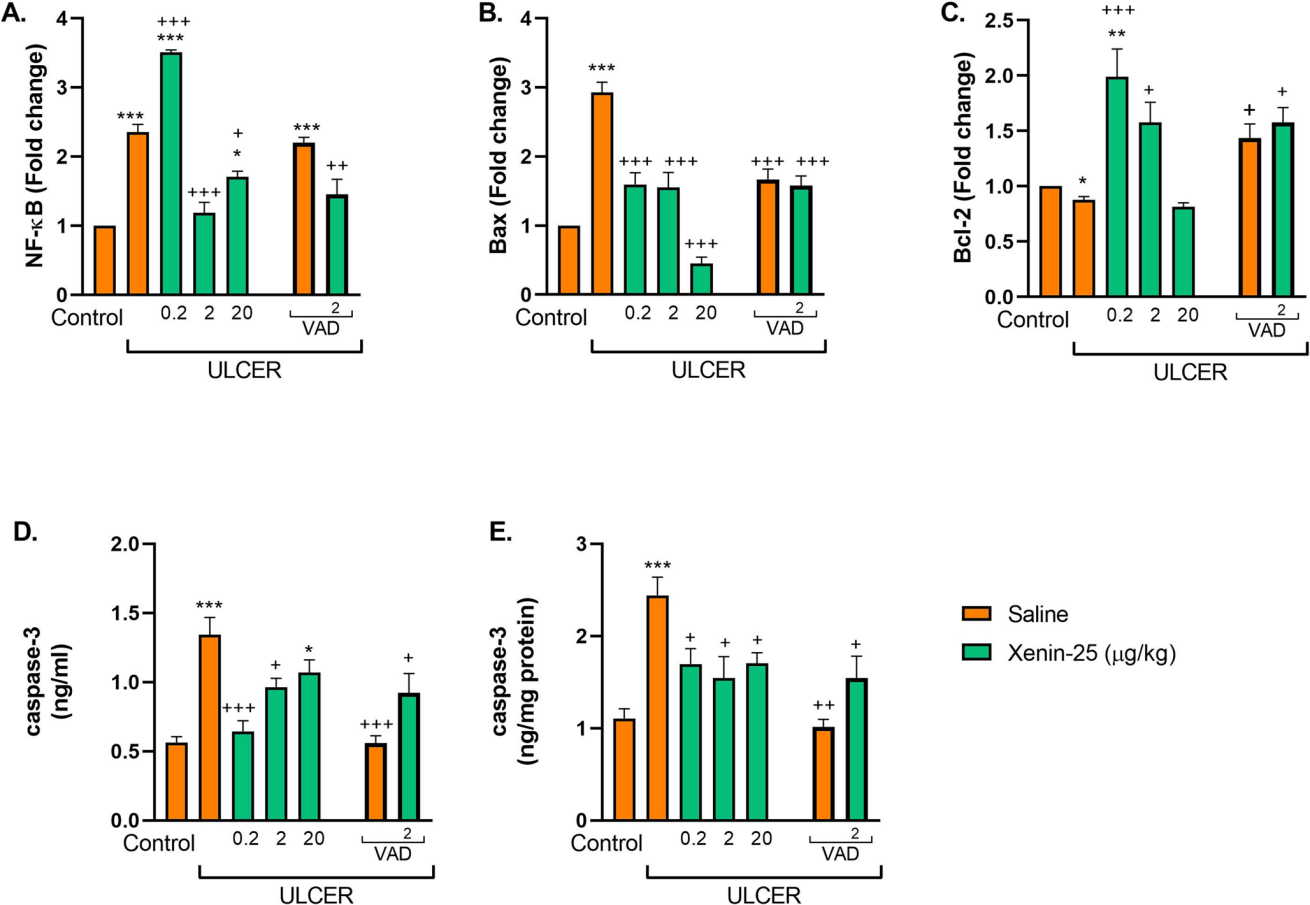
Effects of Xenin-25 on gastric mRNA expression of apoptotic markers and nuclear factor kappa B (NF-κB), and levels of caspase-3 in the indomethacin-induced ulcer rat model. *n* = 3 per each group. (**A**) Nuclear factor kappa B (NF-κB), (**B**) Bcl-2-associated X protein (Bax), (**C**) B-cell lymphoma 2 (Bcl-2), (**D**) serum caspase-3, (**E**) gastric caspase-3 levels. *p < 0.05, **p < 0.01, ***p < 0.001 vs. control; +*p* < 0.05, ++*p* < 0.01, +++*p* < 0.001 vs. ulcer group. VAD: Vagal afferent denervation with capsaicin, X-25: Xenin-25

*NF-κB* mRNA expression was significantly increased in the saline-treated ulcer group as compared to the control (*p* < 0.001, Fig. 6A). Interestingly, xenin-25 at 0.2 µg/kg markedly increased *NF-κB* mRNA expression compared to the ulcer group (*p* < 0.001), whereas higher doses (2 and 20 µg/kg) led to a significant downregulation (*p* < 0.05 − 0.001).

*Bax* mRNA expression was significantly increased in the saline-treated ulcer group as compared to the control (*p* < 0.001), while all doses of xenin-25 reduced its expression, with the strongest effect observed at the highest dose (*p* < 0.001; Fig. 6B). Compared to the control, *Bcl2* expression was decreased in the ulcer group (*p* < 0.05), but was upregulated by the 0.2 and 2 µg/kg doses of xenin-25 (*p* < 0.001, 0.05, respectively; Fig. 6C). Caspase-3 levels in the gastric tissue and serum were elevated in the saline-treated ulcer group, while xenin-25 at all doses significantly reduced caspase-3 levels (*p* < 0.05 − 0.001, Fig. 6D and E).

### Impact of vagal afferent fibers in the gastroprotective effects of xenin-25

When evaluating the contribution of vagal afferent fibers in the gastroprotective effects of xenin-25, our findings indicate that macroscopic and microscopic ulcer scores were similar between the xenin-25-treated rats with intact vagal afferent fibers and those with vagal afferent denervation (VAD) (Figs. 1 and 2). Lesion length, however, was substantially increased in the denervated group (*p* < 0.05), verifying that vagal afferent signaling contributes to xenin-25–mediated reduction in mucosal injury size. VAD did not modify the ability of xenin-25 in preserving GSH levels, whereas it markedly weakened the effect of xenin-25 in reducing MDA levels and suppressing MPO activity (*p* < 0.001, Fig. 4). The effects of xenin-25 on serum TNF-α and IL-6 levels, as well as *NF-κB* expression, were not significant (Figs. 5 and 6). Similarly, the levels of the apoptotic markers *Bax*,* Bcl2*, and caspase-3 were comparable between the xenin-25-treated ulcer groups with intact and denervated vagal afferent fibers. On the other hand, macroscopic scores, lesion length, and histopathological scores in the VAD group were lower than in the ulcer group (*p* < 0.01 − 0.001). MDA levels were decreased, and GSH levels were increased compared to the ulcer group (*p* < 0.05 − 0.001), whereas MPO levels were elevated (*p* < 0.001). TNF-α and IL-6 levels in both gastric tissue and serum were reduced in the VAD group relative to the ulcer group (*p* < 0.05 − 0.001). *NF-κB* expression was lower than in the ulcer group, but the difference was not statistically significant. Additionally, *Bax* and caspase-3 levels were decreased, whereas *Bcl2* expression was increased compared to the ulcer group (*p* < 0.05 − 0.001).

## Discussion

The present study demonstrates, for the first time, that xenin-25 exerts significant gastroprotective effects against indomethacin-induced acute gastric ulceration through its antioxidant, anti-inflammatory and anti-apoptotic properties. The protective actions of xenin-25 were evident across multiple biological levels, including reductions in gastric lesions, oxidative stress markers (MDA, GSH), inflammatory (TNF-α, IL-6, *NF-κB*), and key apoptotic mediators (*Bax*,* Bcl2*, caspase-3). Our results also showed that the gastroprotective actions of xenin-25 were maintained even in the absence of vagal afferent fibers.

Nonsteroidal anti-inflammatory drug (NSAID)-induced gastric ulcers are primarily driven by excessive production of reactive oxygen species (ROS), mucosal inflammation, and increased epithelial apoptosis [29–31]. Consistent with previous findings, indomethacin administration in the present study resulted in significantly elevated gastric MDA and MPO levels, accompanied by a marked depletion of GSH, indicating pronounced lipid peroxidation, oxidative damage and neutrophil infiltration. Treatment with xenin-25 effectively counteracted these alterations, restoring antioxidant capacity, reducing inflammatory cell activity, and reducing gastric damage. A previous study demonstrated that chronic administration of the xenin-25 analog, xenin-25[Lys(13)PAL], in high-fat diet-fed mice suppressed neuroinflammation and oxidative stress by downregulating the NF-κB and TLR4 pathways in the brain [21]. In addition to its anti-inflammatory properties, xenin-25 exerts important physiological effects on gastrointestinal ion transport. Kuwahara et al. (2022) demonstrated that the minimal active domain of xenin-25 can induce Cl⁻/HCO₃⁻ secretion in the rat ileum [20]. Consistent with these findings, Kaji et al. (2017) demonstrated that xenin-25 enhances the secretion of duodenal HCO₃⁻ and Cl⁻ by activating neurotensin receptor-1 (NTS1) found on both intrinsic and extrinsic afferent nerves [32]. This neural activation triggers the release of substance P and serotonin (5-HT), thereby enhancing mucosal ion transport and reinforcing epithelial protection. Bicarbonate secretion is crucial for gastric mucosal defense as it neutralizes luminal acid, maintains surface pH, and aids in epithelial repair [33].

Gastric ulcer development is primarily mediated by inflammatory processes, characterized by increased levels of proinflammatory cytokines [34, 35]. These mediators compromise mucosal integrity and hinder tissue repair, further exacerbating their effects by NSAID administration. Xenin-25 treatment markedly reduced both gastric and serum levels of the proinflammatory cytokines TNF-α and IL-6, supporting its anti-inflammatory effect. This suppression was also reflected at the transcriptional level, as *NF-κB* mRNA expression was significantly downregulated in xenin-25-treated groups. While higher doses (2 and 20 µg/kg) showed consistent inhibition, the lowest dose (0.2 µg/kg) unexpectedly increased *NF-κB* expression. Previous studies have reported that xenin-25 is crucial for regulating insulin secretion, enhancing the insulinotropic effects of GIP, and improving glucose homeostasis [36, 37]. The anti-inflammatory effects of xenin-25 may extend beyond metabolic regulation, modulating cytokine production and neuroimmune activation, and supporting its potential in treating conditions with excessive inflammation [21, 36]. Previous studies have shown that capsaicin-sensitive vagal afferent fibers protect against gastric ulceration by regulating oxidative and inflammatory responses [38, 39]. Specifically, TRPV1-positive vagal afferents have been implicated in reducing oxidative stress and mucosal injury by modulating proinflammatory signaling pathways [40, 41]. However, in our study, ulcer-induced VAD group demonstrated marked ulcer healing, accompanied by reduced inflammation and apoptosis, and exhibited increased neutrophil infiltration compared with the intact VAD ulcer group. However, our cytokine responses revealed that the anti-inflammatory activity of xenin-25 in afferent denervated gastric tissue was similar to that of the gastroprotective effect of VAD per se, suggesting that the gastroprotection offered by VAD and xenin-25 do not have synergistic effects.

Apoptotic imbalance plays a pivotal role in the pathogenesis of gastric ulcers, contributing to mucosal degradation and delayed healing [42]. In the present study, indomethacin-induced ulceration led to a significant increase in pro-apoptotic Bax expression and a concomitant decrease in anti-apoptotic Bcl-2, consistent with enhanced apoptotic cell signaling in gastric tissue. Xenin-25 treatment effectively reversed these changes by downregulating *Bax* and upregulating *Bcl2* expression, indicating a shift towards cell survival signaling. Additionally, caspase-3, a key executioner of apoptosis, was markedly elevated in the serum and gastric tissue of the saline-treated ulcer group, but was significantly decreased in xenin-25-treated ulcer groups, further supporting its anti-apoptotic action. These molecular findings were corroborated by histological evidence, which demonstrated substantial preservation of mucosal integrity, reduced epithelial shedding, and diminished inflammatory infiltration in the xenin-25-treated groups. These results indicate that xenin-25 modulates inflammatory pathways and preserves mucosal structure by attenuating apoptosis at both transcriptional and protein levels. The anti-apoptotic effect of xenin-25 observed in our study is supported by the findings of Tanday et al. (2021). In that study, sub-chronic administration of the long-acting xenin-25 analogue [Lys^13PAL] in both high-fat diet-fed and streptozotocin-induced insulin-deficient mice partially or fully restored β-cell areas, and this improvement was closely associated with a marked reduction in β-cell apoptosis [43]. On the other hand, vagal afferent denervation has not significantly altered the anti-apoptotic effects of xenin-25 in the ulcer groups, because *Bax* and *Bcl2* expression levels and caspase-3 concentrations in the xenin-25-treated group with VAD, were comparable to those observed in the xenin-25-treated ulcer group with intact vagal afferents, indicating that the anti-apoptotic effect of xenin-25 is mainly independent of vagal afferent signaling.

In addition to their metabolic functions, neural afferent circuits—especially those involving the vagus nerve—are increasingly recognized as crucial regulators of mucosal integrity, localized inflammation, and responses to tissue injury [44]. Recent evidence indicates that vagal input plays a key role in epithelial repair and immune regulation, establishing it as a vital component of gut-brain communication [45–47]. In NSAID-induced gastric ulcers, disruption of vagal afferents may significantly compromise host defense mechanisms that usually protect the gastric mucosa [48, 49]. Capsaicin-sensitive vagal afferent fibers contribute considerably to preserving gastric mucosal homeostasis by initiating local protective mechanisms [11]. As our study’s findings indicate, afferent fiber denervation by capsaicin or vagal nerve stimulation can protect against gastric damage, whereas ablation of the vagal nerve exacerbates mucosal injury and gastric dysfunctions [38, 49, 50]. Accordingly, high-frequency electrical stimulation of the left cervical vagus nerve has attenuated oxidative stress by enhancing superoxide dismutase activity and reduced apoptosis via modulation of the Bax/Bcl-2 pathway in the myocardial ischemia–reperfusion model [51]. Xenin-25 retained its anti-apoptotic and anti-inflammatory actions in VAD animals, suggesting that the gastroprotective effects of xenin-25 can also proceed via peripheral mechanisms that are independent of vagal afferents [52]. A key limitation of this study is that we did not investigate the specific receptor pathways involved in the observed effects of xenin-25. In particular, the potential role of neurotensin receptor 1 (NTSR1) and its downstream signaling remains unexplored. Additionally, the effects of xenin-25 were evaluated only in the acute phase; therefore, its long-term efficacy remains to be determined. Moreover, a single effective dose of xenin-25 was tested in the VAD model, but assessing different doses in combination with VAD could provide a more comprehensive understanding of dose-dependent interactions.

Our findings report for the first time that xenin-25 exerts strong protective effects against NSAID-induced acute gastric injury, likely through the modulation of oxidative stress, inflammation, and apoptosis. Notably, we also demonstrated that VAD itself confers a protective effect. Importantly, the gastroprotective actions of xenin-25 persist even in the absence of vagal afferents, indicating that its effects are largely independent of vagal signaling. Furthermore, xenin-25 did not exhibit a synergistic interaction with VAD, suggesting that both interventions may act through similar or overlapping mechanisms.

## Data Availability

Data will be made available on request via the corresponding author.
